# The Institutional Worker

**Published:** 1920-11-27

**Authors:** 


### INSTITUTIONAL WORKER

**Being a Special Supplement to "The Hospital"**

### OUR BUREAU OF INFORMATION.

### Rules for Correspondents

1. Every letter must be accompanied by the coupon to be cut from the back cover (inside page) of THE HOSPITAL, current issue, and must contain the name and address of the correspondent with pseudonym for publication if desired. Replies by post cannot be given save under exceptional circumstances at the Editor's discretion.

2. Letters from Approved Homes in reply to special needs published in the Bureau should state terms and full particulars, and be sent prepaid under cover to the Editor of the Bureau with name written across coupon for identification.

3. Proprietors of Homes which have not yet been entered on the List of Approved Homes, but have spare accommodation likely to suit special needs, are invited to write for an application form for registration. The fee for registration, which includes two announcements of the Home in the Bureau and other privileges, is 10s.

4. All communications to be addressed to the Editor of THE HOSPITAL, 28 Southampton Street, Strand. London. W.C. 2, and marked "Bureau of Information."

### INSTITUTION FACTS AND FIGURES.

### The Question Box.

#### PATIENTS' WAITING-LISTS.

THE question was:

**Describe a simple method of recording names of patients awaiting admission, showing at a glance their proper turn for admission, having regard both to period of waiting and the medical urgency of their respective cases.**

**"Odd No." replies:—**

After a patient has been examined by the medical man and his diagnosis obtained, unless treatment can be given at once, his case is classified as being one of the following "grades" awaiting admission to the hospital for treatment, viz.: "Emergencies," "urgent," "soon," and "ordinary." "Emergencies" constitute those patients who are in desperate straits and whose lives are in actual jeopardy. Such patients require treatment at once—a delay of even a few hours may have fatal consequences. These cases naturally should not be included in the waiting-list. "Urgent" signifies a less serious condition, but one which cannot wait more than a few days for admission. "Soon" is meant to include all patients who, while undoubtedly suffering pain and inconvenience, are not in actual immediate danger of losing their lives or permanently impairing their general health through a delay in treatment of a week or so. "Ordinary" cases comprise milder conditions which do not require immediate treatment to arrest more serious developments, and which could wait (without serious injury to the patient) for a period extending up to six months. Such a "grade" might include cases of simpie hernia, slight hæmorrhoids, fistula, varicose veins, and similar ailments, which, provided reasonable precautions are taken by the patient, would not lead to serious consequences if treatment should be delayed for a month or two.

THE IMPORTANCE OF CLASSIFICATION.

Too great care cannot be bestowed on a proper classification of the condition of each patient by the consultant officer, and it will be frequently found necessary to reconsider the "grading" in the light of subsequent examinations in the case of patients whose conditions become more serious before admission has been secured. The question of admission in order of date, or length of time a patient has been on the waiting-list, is one for arrangement according to the type of patients treated, number of patients on the list, etc. In the case of a large general hospital in an industrial district it has been found impracticable to work on the basis of "date of first attendance" except in the case of "ordinary" patients. Persons in each of the other categories require attention at the first possible moment, having regard only to the relative urgency of each case from a medical viewpoint. With "ordinary" patients the time principle may be adopted with advantage. Should any patient in this category need earlier attention than another (who may have waited a longer period), by reason of more serious developments of disease, it is the duty of the consultant officer to re-grade him "urgent" or "soon," according to the urgency of the case.

THE NECESSARY REPORTS.

On examination of the patient for the first time the consulting officer should enter on a card, marked with the date, name, address, age, sex, etc., of the patient, his diagnosis and classification. This card would be retained by the patient for subsequent attendances, after it has been handed to an officer in charge of the "Register of Patients Awaiting Admission" for the full details to be written up therein. The book should be ruled to provide accommodation for the information supplied on the cards, together with two extra columns to record date of sending for patient and date of actual admission. This book should be subdivided by tabbed leaves bearing the names of each consulting officer, so that only the actual patients of one particular consultant appear in each list. This allocation of all patients under their individual consultants is of the utmost importance to the welfare of the patients and the smooth and efficient running of the hospital. If the whole of the patients are pooled in a general list each consultant loses a large amount of personal interest in them, and there is a tendency for the list to grow unnecessarily long. On the other hand, if each officer concerned has an individual list (which may itself be divided for ease of handling into male, female, and children) he has a ready means of reviewing the whole of the patients under his care. As the patients are written for the date of admission is entered in the book and the name ticked off—this maintaining an accurate record. If the size of the hospital warrants it, two separate books may be kept—one "medical" and the other "surgical."

A LIVE LIST.

It should be the duty of the officer in charge of this book to inquire by letter, or other means, if the patient is still requiring treatment when a considerable period elapses before his admission. If this is done every two months it will keep the list fresh. If this is not done the work of the institution may be hampered and the early admission of urgent cases jeopardised.

Unless the waiting-list assumed voluminous proportions, it would be well for the officer in charge to make himself personally acquainted with the requirements of each patient as far as possible, and if a keen eye is kept on new patients admitted a reliable list may be easily maintained, instead of what is too often a skeleton in the cupboard with no really authentic records to support its existence.

(*Continued on p. 2, col. 3.*)

### Question for November.

### A NURSES' LIBRARY.

Describe fully the most practical way of forming and running a nurses' library suitable for probationers, staff nurses, and sisters. The description should include an outline of necessary rules, the administration, the selection of books, finance, and a method for keeping the library up to date and disposing of old books.

**(A minimum payment of 10s. 6d. will be made for each published answer.)**

RULES.

The following rules must be observed:—

1. Contributions must be written on one side of the paper. Conciseness and terseness are desirable features. The MS. must bear the name and address of the sender and be accompanied by coupon to be cut from the back cover (inside page) of the current issue of The Hospital. A pseudonym must be chosen if the name is not to be published.

2. Contributions must be addressed to the Editor of The Hospital Institutional Worker Supplement, 28 & 29 Southampton Street, Strand, London, W.C. 2, should reach him before the end of the current month, and be marked in the left-hand corner "Facts and Figures."

### QUESTIONS INVITED.

In connection with our Question Box, we offer every month five shillings for the best question sent in for consideration. The questions may be on any institutional subject and concern any department, from the secretary's to the porter's, or the matron's to the domestic staff; the one essential is that they must be practical and deal with points that have a definite relation to hospital or institutional administration, upkeep, finance, or management. Points relating to artificers' work, housekeeping, the laundry, out-patients, and other practical matters will be welcome. It is hoped that thus, when difficult or doubtful points arise in the course of their work, institutional workers will be encouraged to put them in the form of a question and send them to the Editor, The Hospital Bureau of Information, 28 Southampton Street, Strand, W.C. 2, marked "Question Box."

The best questions will be published in due course and our readers will be given the opportunity of answering them. Thus all institutional workers may be able to co-operate with the view of helping the work and smoothing the difficulties of each other. In short, we want the Question Box of the *Institutional Worker* to be freely used by every worker habitually.

### ENQUIRIES AND ANSWERS.

### *THE SICK AND IN NEED.*

**Home for Aged Poor.**

The aged couple might possibly obtain the accommodation they require in the Homes for the Aged Poor. Write to the Secretary at 42-44 St. James' Square, W. 11. They must not possess a joint income of less than 15s. or more than 25s. weekly. The Secretary will give you all other necessary information.—K. A. W.

**Pension for Distressed Gentlewoman.**

Write to the Secretary of the Distressed Gentlefolks' Aid Association, 75 Brook Green, W., and ask if they can assist you in your destitution. Give full details of your circumstances when writing.—Distressed.

**Home for Invalid Gentlewoman.**

We very much regret that we know of no suitable home in the localities you mention for the invalid gentlewoman. We give you the address of two homes that you might write to on her behalf—one in Highbury, London, and one in Windsor. Home for Confirmed Invalids, 36 Aubert Park, Highbury, London, N. Inmates pay not less than 25s. per week. Application should be made to the Matron at the home. St. Andrew's Convalescent Hospital, Clewer, near Windsor. A few incurable patients are received at this home upon payment of from 10s. weekly. Apply to the Sister Superior. If you will let us know what weekly sum your friend can pay we will appeal to our approved homes through these columns, who might possibly give her the accommodation she requires. We make no charge for queries answered through these columns.—C. M. J.

**Help for Poor Woman During Confinement.**

If you appeal to the Resident Surgeon at the Western General Dispensary, Cosway Street, Marylebone Road, N.W., he will no doubt provide the necessary help during the poor woman's confinement.—N.W. District.

### *EMPLOYMENT AND TRAINING.*

**Training in Nursing Tropical Diseases.**

You will probably gain all the experience you need in the nursing of tropical diseases at one of the following institutions:—London Hospital for Tropical Diseases, Endsleigh Gardens, Euston Road, N.W. 1; Royal Southern Hospital, Liverpool; or the Royal Albert Dock Hospital, E. 16. Enclose a stamped addressed envelope for reply when writing to the Matron of the individual hospitals.—Margaret.

**Present Rate of Pay for Army Nurses.**

The present rate of pay for staff nurses in Queen Alexandra's Imperial Military Nursing Service is £60, rising by yearly increments of £2 10s. to £65. Write to the Matron-in-Chief, Q.A.I.M.N.S., War Office, Whitehall, S.W. With regard to your query of the Territorial Force Nursing Service, we suggest that you apply for information to the Matron-in-Chief, 80 Pall Mall, London, S.W. 1.—Penelope.

**Society for Welfare Workers.**

Write to the Secretary of the Welfare Workers' Institute, 11 Adam Street, Adelphi, London, W.C., and ask if they can help you. The address of the Royal Institute of Public Health is 37 Russell Square, W.C.—Highfield.

### *MISCELLANEOUS.*

**Where to Obtain Ampoules of Phenolsulphonphthalein.**

You can obtain ampoules of phenolsulphonphthalein of Messrs. Allen and Hanburys, Vere Street, London, W. These ampoules are their own manufacture. The price is 6s. per dozen ampoules. A coupon should be enclosed with each inquiry; we cannot give postal replies.—D. J. F.

**Midwifery Wall Diagrams**

Messrs. H. K. Lewis & Co., Ltd., of 136 Gower Street, London, W.C. 1, will supply you with midwifery wall diagrams suitable for lectures. These particular diagrams are by Dr. Victor Bonney, and can be obtained in a series containing 24 sheets, 26 by 40, at 2s. 6d. a sheet; or the set for 52s. 6d.; on linen 75s.—Medical Officer of Health.

**Portable Electric Serving Table.**

The Morandi-Proctor Co., 86 Washington Street, Boston, Mass., have designed an excellent portable electric serving table for use in hospital wards. The table is electrically heated by attaching it to an electric wall socket. If you write to Messrs. Morandi-Proctor they will forward you full particulars and prices.—McC.

### Patients' Waiting-Lists.

(*Continued from p. 1.*)

If it is possible to extend an already existing card system the above method might be considerably simplified by filing the actual examination cards (on which are entered the date, patient's name, etc., diagnosis, and classification), behind guide cards printed with the name of the consultant. The advantage of the card system lies in the fact that the card can be extracted when the patient is admitted, thus keeping only a list of live cards, and obviating the use of a bound book and consequent ticking off of names. However good the system may be, it can only be efficiently worked if the person responsible for the records takes an interest in the patients and uses his powers to bring to the notice of the consulting officers any cases of delay which might be avoided. After all, the ideal hospital has no waiting-list.

